# Supplementary material for: Exportin 1‐mediated nuclear/cytoplasmic trafficking controls drug sensitivity of classical Hodgkin's lymphoma
Source: Mol Oncol. 2023 Apr 21;17(12):2546–64. doi: 10.1002/1878-0261.13386 (PMC10701774; doi:10.1002/1878-0261.13386)
Supplement: Supplementary file 1 — Fig. S1. Schematic representation of the CRISPR‐Cas9 knockout strategy set up for MedB1 cells. Fig. S2. Sequential extraction of cytoplasmic and nuclear proteins. Fig. S3. Degradation of the XPO1 protein in cHL expressing the wt or the mutant XPO1 protein. Fig. S4. Western blots for controlling the efficacy of ibrutinib and selinexor treatments. Fig. S5. U2940 cells, KS/KAS, and EAS/ES derivatives display the same proliferation curve. Fig. S6. Characterization of the in vivo/in ovo model of cHL cell line engraftment. Fig. S7. Schedule for cHL cell line engraftment on CAM and treatments. Fig. S8. Effects of drugs on L428 tumor weights. Fig. S9. The selinexor/ibrutinib combination is synergistic on cHL cells. Fig. S10. DNA‐binding activity of NFκB proteins in cHL. Fig. S11. Indirect immunofluorescence analysis of NFκB proteins in cHL cells. Fig. S12. Western blot analysis of p‐p65 expression in cHL cell lines. Fig. S13. Original blots for Fig. 2C. Table S1. Antibodies used in this study. Table S2. B‐cell lymphoma cell line characteristics. Table S3. Sequences of crRNA used in the CRISPR‐Cas9 strategy. Table S4. Sequences of the primers used for XPO1 PCR amplification and Sanger sequencing. Table S5. Sequences of the primers designed for qRT‐PCR studies. Table S6. In ovo effects of drug treatment on cHL cell line growth. Table S7. Effects of drug treatment on L428 tumor weight (mg)Figure legends. Table S8. Effects of drug treatment on L428 tumor volume (mm3). Table S9. Fluorescence intensity of nuclear NFκB proteins in cHL. Table S10. Fn/c calculated from the four cHL cell lines following selinexor treatment. Table S11. Fluorescence intensity of nuclear nucleophosmin and survivin in SUPHD1 and UHO1 cells treated with selinexor. Table S12. Fn/c calculated from L428, L1236 and UHO1 cell lines following importazole treatment. Table S13. Fluorescence intensity of cytoplasmic p65 protein in cHL. Table S14. Number of IPO1/XPO1 or IPO1/NFκB dimers in cHL cells. [file MOL2-17-2546-s001.pdf]

Exportin 1-mediated nuclear/cytoplasmic trafficking controls drug sensitivity of classical Hodgkin lymphoma

Mélody Caillot, Hadjer Miloudi, Antoine Taly, Nuria Profitós-Pelejà, Juliana C. Santos,

Marcelo L. Ribeiro, Elsa Maitre, Simon Saule, Gaël Roué, Fabrice Jardin, Brigitte Sola

Supplementary data

Table S1. Antibodies used in this study

Protein	Reference	Species	Origin	Dilution (assay)
β-actin	sc-47778<br>#4970	Mouse<br>Rabbit	Santa Cruz Biotech.<br>Cell Signaling Tech.	1/1 000 (WB)<br>1/1 000 (WB)
p-BTK	#5082	Rabbit	Cell Signaling Tech.	1/1 000 (WB)
Cl. caspase 3	#9664	Rabbit	Cell Signaling Tech.	1/400 (IHC)
ENO1	GTX113179	Rabbit	GenTex	1/5 000 (WB)
GAPDH	sc-137179	Mouse	Santa Cruz Biotech.	1/100 (WB)
IPO1	ab2811	Mouse	abcam	1/1 000 (PLA)
Ki67	GA626	Mouse	Dako	1/150 (IHC)
p65	#8242<br><br>ab32536	Rabbit<br><br>Rabbit	Cell Signaling Tech.<br><br>abcam	1/1 000 (WB)<br>1/100 (IF)<br>1/100 (IF)<br>1/50 (PLA)
pSer536-p65	#3033	Rabbit	Cell Signaling Tech.	1/1 000 (WB)
PARP	ab191217	Rabbit	abcam	1/ 1000 (WB)
*NFκB1 p105/p50	#3035<br>ab131546	Rabbit<br>Rabbit	Cell Signaling Tech.<br>abcam	1/1 000 (WB)<br>1/100 (IF)
*NFκB2 p100/p52	#3017<br>ab175192	Rabbit<br>Rabbit	Cell Signaling Tech.<br>abcam	1/1 000 (WB)<br>1/100 (IF)
Nucleophosmin (NPM)	#10530	Mouse	abcam	1/200 (IF)
c-REL	sc-6955	Mouse	Santa Cruz Biotech.	1/200 (WB)<br>1/50 (IF)<br>1/50 (PLA)
RELB	#4922<br>HPA040506	Rabbit<br>Rabbit	Cell Signaling Tech.<br>Sigma	1/1 000 (WB)<br>1/20 (IF)
Survivin (BIRC5)	#469	Rabbit	abcam	1/200 (IF)
XPO1	sc-74454<br><br><br>A300-469A<br>PLA-0109	Mouse<br><br><br>Rabbit<br>Rabbit	Santa Cruz Biotech.<br><br><br>Bethyl Laboratories<br>Sigma-Aldrich	1/1 000 (WB)<br>1/50 (IF)<br>1/50 (PLA)<br>1/500 (WB)<br>1/100 (IF)

*The anti-NFκB1 and anti-NFκB2 Abs revealed the p105 and p100 precursors as well as the p50 and p52 mature proteins.

Abbreviations: Cl, cleaved; IF, indirect immunofluorescence; IHC, immunohistochemistry; PLA, proximity ligation assay; WB, western blot.

Table S2. B-cell lymphoma cell lines characteristics

**Cell line**	**Pathology**	**DSMZ***	**Cellsaurus**	**COSMIC_ID**	***XPO1***
KMH2	cHL	ACC-8	CVCL_1330	1432041	wt ampl. (5)
L428	cHL	ACC 197	CVCL_1361	1714161	wt ampl. (7)
L1236	cHL	ACC 530	CVCL_2096	1289701	wt ampl./E571K (6)
SUPHD1	cHL	ACC 574	CVCL_2208	1432043	wt/E571K
UHO1	cHL	ACC 626	CVCL_2220	-	wt/E571K dupl.
UHO1Δmut	cHL				wt/del
K1106	PMBL	-	CVCL_1821	2479248	wt dupl.
MedB1	PMBL	-	CVCL_A649	-	wt/E571K
MedB1Δwt	PMBL				del/E571K
U2940	PMBL	ACC 634	CVCL_1897	243272	wt
EAS/ES	PMBL				wt/C528S + E571E
KAS/KS	PMBL				wt/C528S + E571K

* cHL and PMBL cells were purchased from DSMZ (Leibniz Institute, Braunschweig, Germany) except Karpas 1106-P (referred here to K1106) and MedB1, a generous gift of Karen Leroy (Institut Cochin, Paris, France). Cells were authenticated by STR profiling (DSMZ). *XPO1* status was reported previously [13,14] and finally confirmed with the data base : https://cellmodelpassports.sanger.ac.uk/. In cell lines with amplified *XPO1*, the copy number is indicated in parentheses.

Abbreviations: ampl., amplification; cHL, classical form of Hogdkin lymphoma; del, deleted; dupl., duplication; PMBL, primary mediastinal B-cell lymphoma; nd, not determined; wt, wild-type.

Table S3. Sequences of crRNA used in the CRISPR-Cas9 strategy

**Target**	**Sequences of oligonucleotides**
XPO1_wt	5’-AAG CUG UUC **GAA** UUC-3’
XPO1_mut	5’-AAG CUG UUC **AAA** UUC-3’

The nucleotides corresponding to wt and mutant (mut) human *XPO1* gene (locus NM_003400) are in bold.

Table S4. Sequences of the primers used for *XPO1* PCR amplification and Sanger sequencing

Forward	5’-TGT GTT GGG CAA TAG GCT CC-3’
Reverse	5’-TGA ACC TGA ACG AAA TGC CTG C-3’

The primer sequences were designed with the primer 3 software (v4.0, //primer3.ut.ee/).

Table S5. Sequences of the primers designed for qRT-PCR studies

Gene	Protein	Sequence 5'-3' (forward and reverse)
BCL2	BCL2	ACA GGG TAC GAT AAC CGG GA<br>GGG CCG TAC AGT TCC ACA AA
BCL2L1	BCLXL	GGG AGG CAG GCG ACG AGT TT<br>CAC AGT GCC CCG CCG AAG GA
CCND2	Cyclin D2	CAC TTG TGA TGC CCT GAC TGA<br>GGC AAG CTT TGA GAC AAT CCA
CCND3	Cyclin D3	CCA TCG AAA AAC TGT GCA TCT ACA<br>CCT CCC AGT CCC GCA ACT
CDK2	CDK2	GGC ACG TAC GGA GTT GTG TA<br>GCC AGT CAC CTC AGC AA
CDK4	CDK4	TGG CTG AAA TTG GTG TCG GT<br>ACG GGT GTA AGT GCC ATC TG
CDK6	CDK6	ACA GAG CAC CCG AAG TCT TG<br>GTA TGG GTG AGA CAG GGC AC
CDKN1A	$p21^{CIP}$	ACC CTA GTT CTA CCT CAG GC<br>AAG ATC TAC TCC CCC ATC AT
CDKN1B	$p27^{KIP}$	GTG CGA GTG TCT AAC GGG AG<br>AGT AGA ACT CGG GCA AGC TG
GAPDH	GAPDH	CTG ACT TCA ACA GCG ACA CC<br>CCC TGT TGC TGT AGC CAA AT
MYC	MYC	GGC AAA AGG TCA GAG TCT GG<br>GTG CAT TTT CGG TTG TTG C
MCL1	MCL1	TCG GCC CGG CGA GAG ATA GG<br>TCC GGG AGT CTG GCG TGA GG
RPLP0	RPLP0	CCA GGC GTC CTC GTG GAA GTG<br>TTC CCG CGA AGG GAC ATG CG

The primer sequences directed against the corresponding genes were designed with the primer 3 software (v4.0, //primer3.ut.ee/). *GAPDH* and *RPLP0* served as internal controls.

Table S6. *In ovo* effects of drugs treatment cHL cell lines growth

Cell line	Treatment (dose)	Number of eggs	Tumor weight (mg) means ± s.d.
**KMH2**	DMSO	5	90.40 ± 15.73
	Ibrutinib (50 µM)	5	23.20 ± 6.30
	Selinexor (5 µM)	5	36.60 ± 6.88
	Ibru (50 µM)/sel (5µM)	5	12.70 ± 4.39
**L428**	DMSO	10	83.30 ± 27.62
	Ibrutinib (50 µM)	12	49.58 ± 27.17
	Selinexor (5 µM)	11	56.36 ± 26.61
**SUPHD1**	DMSO	14	64.71 ± 36.88
	Ibrutinib (50 µM)	17	11.41 ± 6.17
	Selinexor (50 nM)	12	35.08 ± 24.10
	Selinexor (100 nM)	10	16.40 ± 10.26
**UHO1**	DMSO	15	36.33 ± 13.17
	Ibrutinib (20 µM)	13	29.69 ± 17.17
	Ibrutinib (50 µM)	12	15.17 ± 9.52
	Selinexor (20 nM)	12	34.00 ± 15.08
	Selinexor (100 nM)	15	18.47 ± 9.96
	Ibru (20 µM)/sel (100 nM)	10	7.60 ± 3.24

KMH2, L428, SUPHD1 and UHO1 cells were engrafted on the chick embryonic CAM at D9. Treatments started two days layer (D11) with 0.1% DMSO in the control arm, ibrutinib or selinexor at the indicated doses for the two others, each two days until D15. At D16, tumors were removed and weighted. The number of surviving embryos is indicated in the table. The means ± s.d. of tumor weight are reported. The statistics are presented in the Fig. 3B of the main text..

Table S7. Effects of drugs treatment on L428 tumor weight (mg)

	**Vehicle**	**Ibrutinib**	**Selinexor**	**Combo**
Number of mouse	4	3	5	5
Minimum	220.0	45.0	115.0	30.0
Maximum	670.0	242.0	471.0	110.0
Range	450.0	197.0	356.0	80.0
Mean	376.8	154.0	222.0	66.0
s.d.	203.8	105.2	145.1	32.9

L428 (10^7 cells in 100 µl final volume of medium) were engrafted with matrigel (vol/vol) s.c. on the flank of SCID mice. When tumors were detected by palpation, mice were separated in four groups (n = 4 for control and n =5 for the others) and received either vehicle (0.1% DMSO), selinexor (10 mg/kg, twice a week), ibrutinib (25 mg/kg, three times per week) as monotherapies or the combination of drugs. At day 35, mice were sacrificed and tumors removed and weighted. Two mice died unexpectedly during the course of the treatment in the ibrutinib series. To illustrate the high tumor viariability among the mice, the minimum, maximum, and range of tumor weights are indicated in the table as well as the means ± s.d. The *p*-values calculated with the *t*-test, were not statistically different except $p = 0.0112$ for combo vs vehicle and $p = 0.0471$ for combo vs selinexor (see Fig. S8).

Table S8. Effects of drugs treatment on L428 tumor volume (mm^3)

	Vehicle	Ibrutinib	Selinexor	Combo
Number of mouse	4	3	5	5
Minimum	161.8	72.0	102.6	37.5
Maximum	637.1	376.7	492.8	89.2
Range	475.2	304.6	390.2	51.7
Mean	384.1	201.0	208.4	63.9
s.d.	245.5	157.6	164.4	24.8

At the end of the experiment, tumors were measured and their volumes calculated with the formula: volume in mm^3 = 1/2(length × $width^2$) using digital calipers. As before, the volumes of tumor were highly heterogeneous. Due to this heterogeneity and the low number of animal in each series, only the combo vs vehicle *p*-value is statitically diffreent with $p = 0.024$ (see Fig. 3C).

Table S9. Fluorescence intensity of nuclear NFκB proteins in cHL

Cell line		n	Minimum	Maximum	Mean ± s.d.
**L428**	NFκB1 p105/p50	50	7.082	31.46	14.71 ± 4.404
	NFκB2 p100/p52	50	6.806	34.82	20.50 ± 6.499
	p65	50	5.909	53.05	21.52 ± 10.75
	RELB	50	14.62	39.63	26.79 ± 5.915
	cREL	50	1.666	22.27	10.57 ± 6.248
**L1236**	NFκB1 p105/p50	50	4.195	29.09	13.99 ± 5.975
	NFκB2 p100/p52	50	9.555	44.01	26.00 ± 9.491
	p65	50	1.628	47.33	18.46 ± 10.97
	RELB	50	3.378	36.30	21.96 ± 8.915
	cREL	50	0.019	28.54	7.15 ± 7.566
**SUPHD1**	NFκB1 p105/p50	50	9.031	46.37	18.37 ± 7.117
	NFκB2 p100/p52	50	5.311	31.18	13.58 ± 6.488
	p65	50	1.88	15.56	9.096 ± 3.250
	RELB	50	4.731	44.76	24.55 ± 9.659
	cREL	50	0.556	24.03	9.677 ± 6.609
**UHO1**	NFκB1 p105/p50	50	4.734	23.32	12.77 ± 4.300
	NFκB2 p100/p52	50	0.8740	35.94	13.16 ± 8.026
	p65	50	2.402	49.63	11.93 ± 8.083
	RELB	50	12.83	40.33	22.70 ± 5.566
	cREL	50	4.213	24.60	10.81 ± 4.387

Cultured cHL were analyzed by indirect IF for NFκB proteins (NFκB1/p50, NFκB2/p52, p65 (RELA), RELB and cREL) localization and expression. The minimum, maximum and mean of FI for nuclear proteins are indicated in the table as well as the number of individual cells recorded (n) for each experimental condition. The corresponding boxplots are presented in Fig. 4D.

Table S10. Fn/c calculated from the four cHL cell lines following selinexor treatment

**Cell line**		**Fn/c (V)**	**Fn/c (S)**	**n (V)**	**n (S)**	***p*-value**
**L428**	NFκB1 p105/p50	2.13	2.62	103	100	0.0698
	NFκB2 p100/p52	18.21	25.04	100	101	< 0.0001
	p65	5.37	9.22	102	106	< 0.0001
**L1236**	NFκB1 p105/p50	1.49	1.49	101	100	0.9655
	NFκB2 p100/p52	5.41	4.87	102	101	0.2845
	p65	6.25	10.43	100	100	< 0.0001
**SUPHD1**	NFκB1 p105/p50	1.39	1.36	105	100	0.8431
	NFκB2 p100/p52	6.75	7.97	103	96	0.1072
	p65	14.72	14.21	100	100	0.7415
**UHO1**	NFκB1 p105/p50	1.77	1.73	101	100	0.7998
	NFκB2 p100/p52	5.02	5.29	102	98	0.4774
	p65	4.19	3.76	100	100	0.1006

Cultured cells were treated with vehicle (V) or selinexor (S), then analyzed by indirect IF for NFκB proteins localization. The means of calculated Fn/c for each antibody are indicated in the table as well as the number of individual cells recorded (n) for each experimental condition. We calculated the ratio Fn/c with the ImageJ software. An increased Fn/c indicated the nuclear accumulation of the protein. The cooresponding boxplots are reported Fig. 5A. To compare selinexor- vs vehicle-treated cells, the *p*-value (paired *t*-test) was calculated with the PRISM software.

Table S11. Fluorescence intensity of nuclear nucleophosmin and survivin in SUPHD1 and UHO1 cells treated with selinexor

	**Protein**	**Treatment**	**n**	**Minimum**	**Maximum**	**Mean ± s.d.**
**SUPHD1**	NPM	Vehicle	50	0.06	4.90	1.16 ± 1.20
		Selinexor	51	0.11	28.29	11.62 ± 6.99
	BIRC5	Vehicle	50	0.83	39.57	15.71 ± 10.30
		Selinexor	50	4.04	78.96	± 21.09
**UHO1**	NPM	Vehicle	50	1.14	16.66	7.09 ± 3.57
		Selinexor	51	0.08	47.31	16.62 ± 13.35
	BIRC5	Vehicle	50	1.29	42.30	20.45 ± 11.90
		Selinexor	50	4.47	82.62	39.53 ± 22.88

Cultured SUPHD1 and UHO1 cells were treated with vehicle or selinexor (100 nM for 6 h), then analyzed by indirect IF for nucleophosmin (NPM) and survivin (BIRC5) localization. The minimum, maximum and mean of fluorescence intensity (FI) for nuclear NPM and BIRC5 are indicated in the table as well as the number of individual cells recorded (n) for each experimental condition. The corresponding boxplots are presented in Fig. 5B.

Table S12. Fn/c calculated from L428, L1236 and UHO1 cell lines following importazole treatment

Cell line	Protein	Fn/c (V)	Fn/c (I)	n (V)	n (IPZ)	*p*-value
**L428**	p52/NFκB2	18.21	3.25	100	100	< 0.0001
	p65	5.37	2.67	102	102	< 0.0001
	RELB	9.99	1.83	110	101	< 0.0001
**L1236**	p52/NFκB2	5.41	3.27	102	98	< 0.0001
	p65	6.25	1.18	100	100	< 0.0001
	RELB	6.33	4.47	101	100	0.0004
**SUPHD1**	p52/NFκB2	6.752	2.82	103	100	< 0.0001
	p65	14.72	2.07	100	100	< 0.0001
	RELB	5.38	1.72	101	104	< 0.0001
**UHO1**	p52/NFκB2	5.02	2.79	102	99	< 0.0001
	p65	4.19	1.92	100	100	< 0.0001
	RELB	7.12	0.88	100	100	< 0.0001

Cultured cells were treated with vehicle (V) or importazole (I), then analyzed by indirect IF for NFκB proteins localization. The mean of FI for each antibody is indicated in the table as well as the number of individual cells recorded (n) for each cell line. We calculated the ratio Fn/c with the ImageJ software as reported previously. They are presented in the Fig. 5C. The *p*-value (paired *t*-test) was calculated with the PRISM software for the comparison of importazole- vs vehicle-treated cells.

Table S13. Fluorescence intensity of cytoplasmic p65 protein in cHL

Cell line	n	Minimum	Maximum	Mean ± s.d.
**L428**	50	0.166	5.031	1.711 ± 1.129
**L1236**	49	0.217	5.258	1.782 ± 1.155
**SUPHD1**	51	0.260	5.058	1.547 ± 1.094
**UHO1**	51	0.263	5.015	1.478 ± 1.187

Cultured cHL were analyzed by indirect IF for nuclear p65 expression. The minimum, maximum and mean of fluorescence intensity (FI) of cytoplasmic proteins is indicated in the table as well as the number of individual cells recorded (n) for each cell line. The corresponding boxplots are presented in Fig. 6A.

Table S14. Number of IPO1/XPO1 or IPO1/NFκB dimers in cHL cells

Cell line	Protein	n	Minimum	Maximum	Mean ± s.d.
**SUPHD1**	XPO1	100	2	45	11.62 ± 6.98
	p52	100	0	2	0.38 ± 0.89
	p65	99	0	4	0.61 ± 0.62
**UHO1**	XPO1	100	4	31	16.15 ± 6.86
	p52	101	0	3	0.48 ± 0.86
	p65	100	0	28	5.12 ± 5.90

From the PLA assays, the number of red dots representing one dimer was counted in the indicated number of cells (n) with the ImageJ software. The number of the minimum and maximum dots in one cell was recorded, the means as well as s.d. were calculated with the same software.

Supplementary Figures

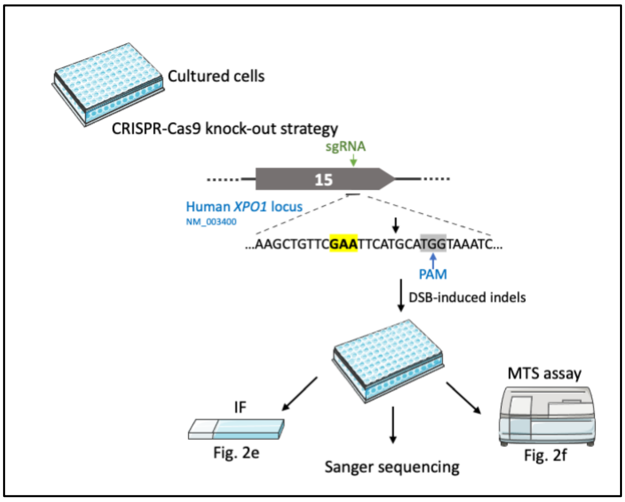

Fig. S1. Schematic representation of the CRISPR-Cas9 knockout strategy set up for MedB1 cells
We used an sgRNA (Table S3, green arrow) that targets the E571 codon (in yellow and bold) to delete the wt *XPO1* allele. To ensure a high editing efficiency, we choose the PAM site (in grey) close to the targeted codon. MedB1 cells were transfected by nucleofection. Two days later, gDNA was purified from edited MedB1 cells, PCR-amplified with the primers described in the Table S4. PCR fragments were purified and sequenced by the Sanger method. Edited cells (Δwt) were maintained in culture and compared to parental cells (p). The expression of XPO1 was analyzed by indirect IF (Fig. 2E). The sensitivity of both cell types toward selinexor and ibrutinib was assessed with an MTS assay (Fig. 2F).

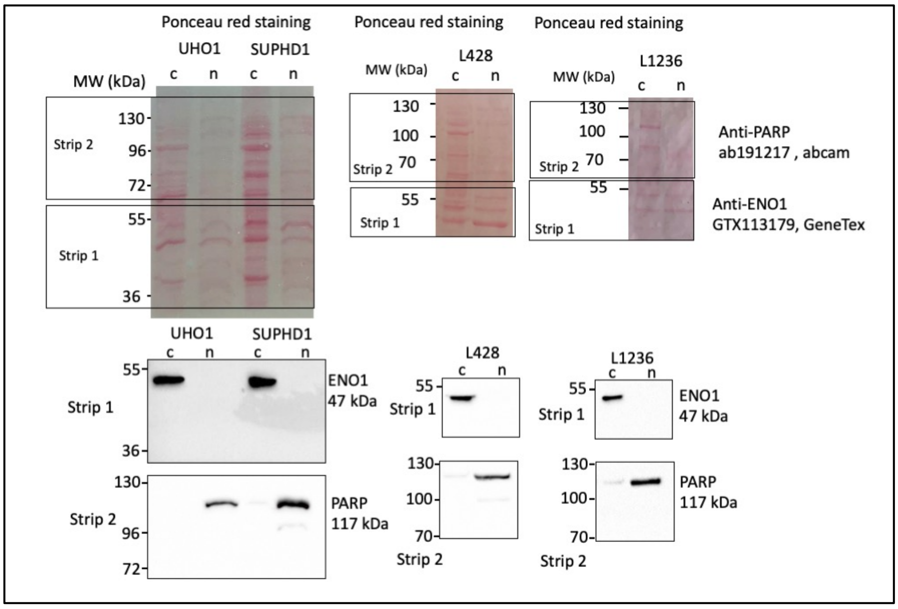

Fig. S2. Sequential extraction of cytoplasmic and nuclear proteins
Cultured cHL cells were harvested. Cytosolic and nuclear extracts were prepared with the NE-PER Nuclear and Cytoplasmic Extraction Reagent (Thermo Scientific) according to the manufacture instructions. Proteins (30 µg) were separated on SDS-PAGE and transferred onto nitrocellulose membrane. Membranes were cut into strips that were incubated with specific Abs recognizing either poly (ADP-ribose) polymerase 1 (PARP1) as nuclear protein or enolase 1 (ENO1) as cytosolic protein (Table S1).

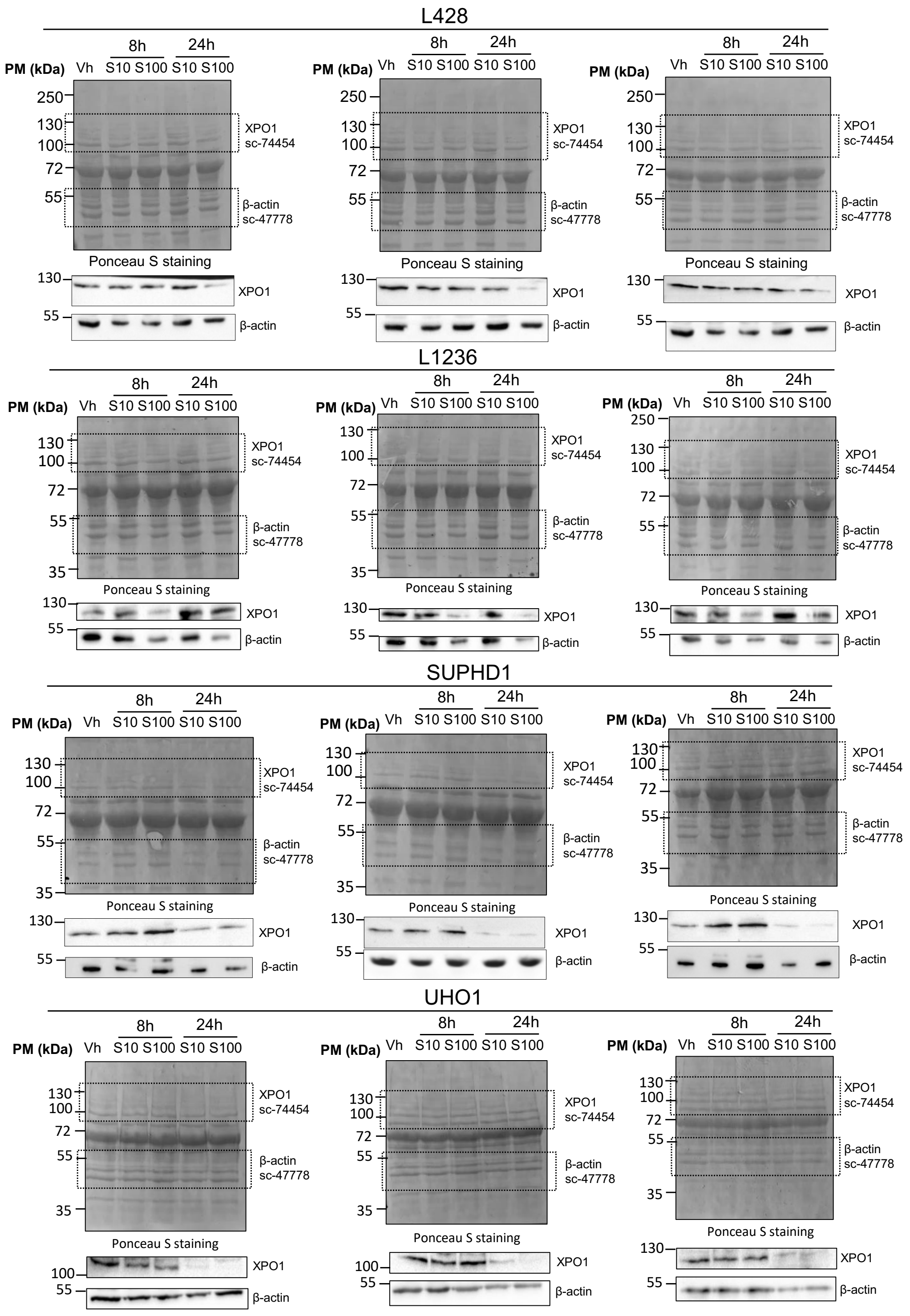

Fig. S3. Degradation of the XPO1 protein in cHL expressing the wt or the mutant XPO1 protein
cHL cell lines were either treated with vehicle- (Vh) or selinexor- (S) (10 or 100 nM) for 8 or 24 h. Whole-cell proteins were purified and separated on SDS-PAGE, transferred onto nitrocellulose membranes, then incubated with the indicated Abs (Table S1). Three independent blots were run and each blot was analyzed by densitometry for the quantification of XPO1 protein level relative to the β-actin level used as an internal control. The level of XPO1 in selinexor-treated cells was then normalized to the one in vehicle-treated cells defined as 1. The values were used to draw the histogram presented Fig. 1E and for the statistical analyses.

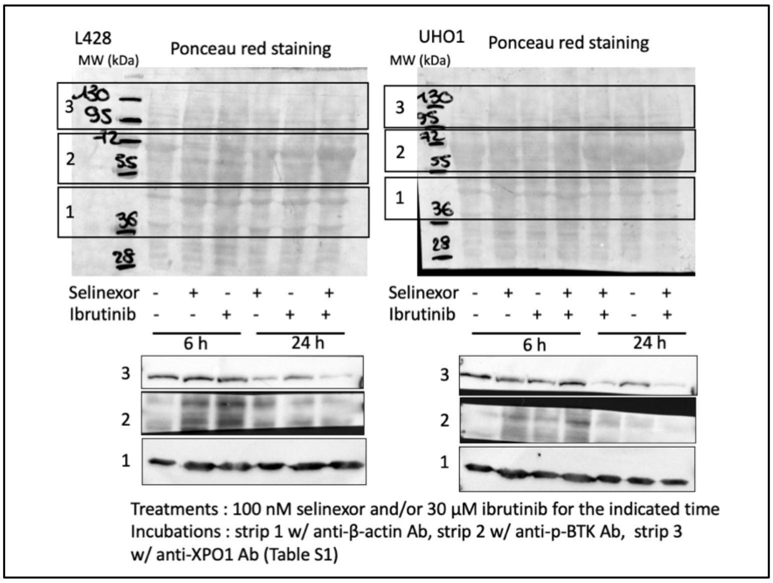

Fig. S4. Western blots for controlling the efficacy of ibrutinib and selinexor treatments

L428 and UHO1 cells were treated with selinexor or ibrutinib or the combination of the two drugs for 6 or 24 h. Whole-cell proteins were purified. Proteins (30 µg) separated by SDS-PAGE, and transferred onto nitrocellulose membranes stained with Ponceau red. According to the scheme, membranes were then cut into strips that were incubated with the indicated Abs (Table S1). Selinexor treatment leads to the degradation of XPO1 in both cell lines 24 h post-treatment, whereas the ibrutinib treatment leads to the disappearance of the phosphorylated form of BTK at the same time. An anti-β-actin Ab served as a control of charge and transfer. Molecular weight markers (MW) were run in the same gels.

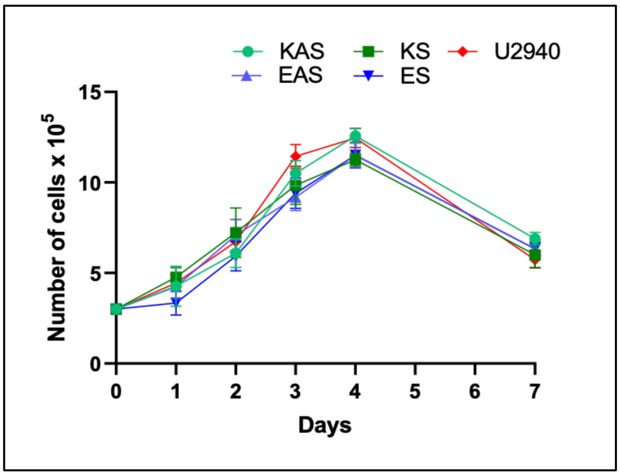

Fig. S5. U2940 cells, KS/KAS and EAS/ES derivatives display the same proliferation curve

Cells were seeded at the density of 3 x 10^5 cells/ml in 24-well plates in complete medium and counted by trypan blue exclusion every day for one week (n = 3). During this period, cells were not diluted. The means of total number of living cells are reported on the graph together with s.d. The experiment was done twice, a representative one is presented.

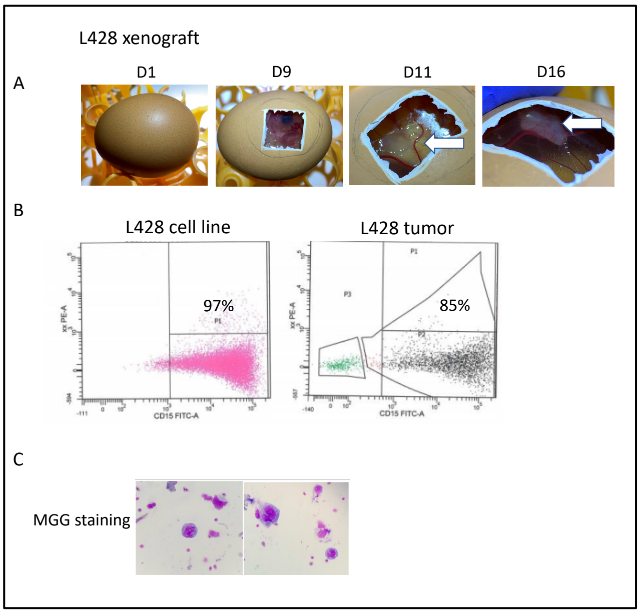

Fig. S6. Characterization of the *in vivo*/*in ovo* model of cHL cell lines engraftment

A, Fertilized eggs (D1, EARL Les Bruyères, Dangers, France) were incubated for nine days at 38°C in an incubator with 55% relative humidity. At that time, the CAM was dropped by drilling a small hole through the eggshell into the air pocket and a small window was cut in the eggshell above the CAM (D9). Cultured tumor cells (10^6) mixed in Matrigel (v/v, 50 µl final volume) were directly added on the CAM beneath. Tumors were visible as soon as two days post-engraftment (D11) and grew all along the duration of the experiment (D16). The vascularized tumor is arrowed in white. **B**, At the end of the experiment (D16), the upper CAM was carefully removed from each egg and the tumor cut out. Tumors were weighted and tumor cells were dissociated with Accutase (Merck) then analyzed for CD15 expression by flow cytometry. Ninety-seven % of cells were CD15-positive in the culture of L428 cells and 85% in the tumor after cell dissociation. **C**, Dissociated tumor cells were cytospinned onto glass slides and stained with May-Grünwald-Giemsa for morphological examination (X 1 000, magnification).

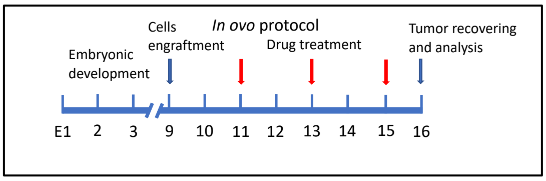

Fig. S7. Schedule for cHL cell lines engraftment on CAM and treatments

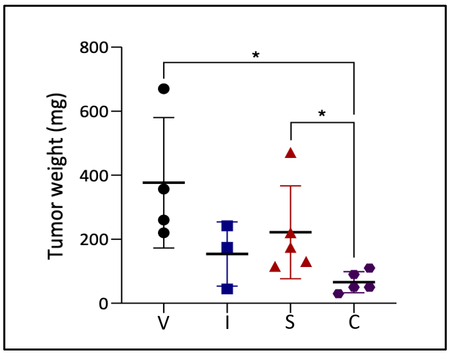

Fig. S8. Effects of drugs on L428 tumor weights

Ten millions of L428 cells mixed in matrigel were engrafted s.c. onto the flank of SCID mice. Eight weeks later, tumor-bearing mice were separated into fours groups receiving : vehicle (n = 4), ibrutinib (I), selinexor (S) or the combination of drugs (C) (n = 5, in each arm). Two mice died in the ibrutinib group. Thirty-five days after the beginning of the treatments, mice were sacrificed and the tumors recovered. They were weightedand the volume of each tumor in each treatment arm is reported in the graph together with the statistical analysis. * $p < 0.05$ with the *t*-test. A similar graph was drawn with the tumor volumes (Fig. 3C).

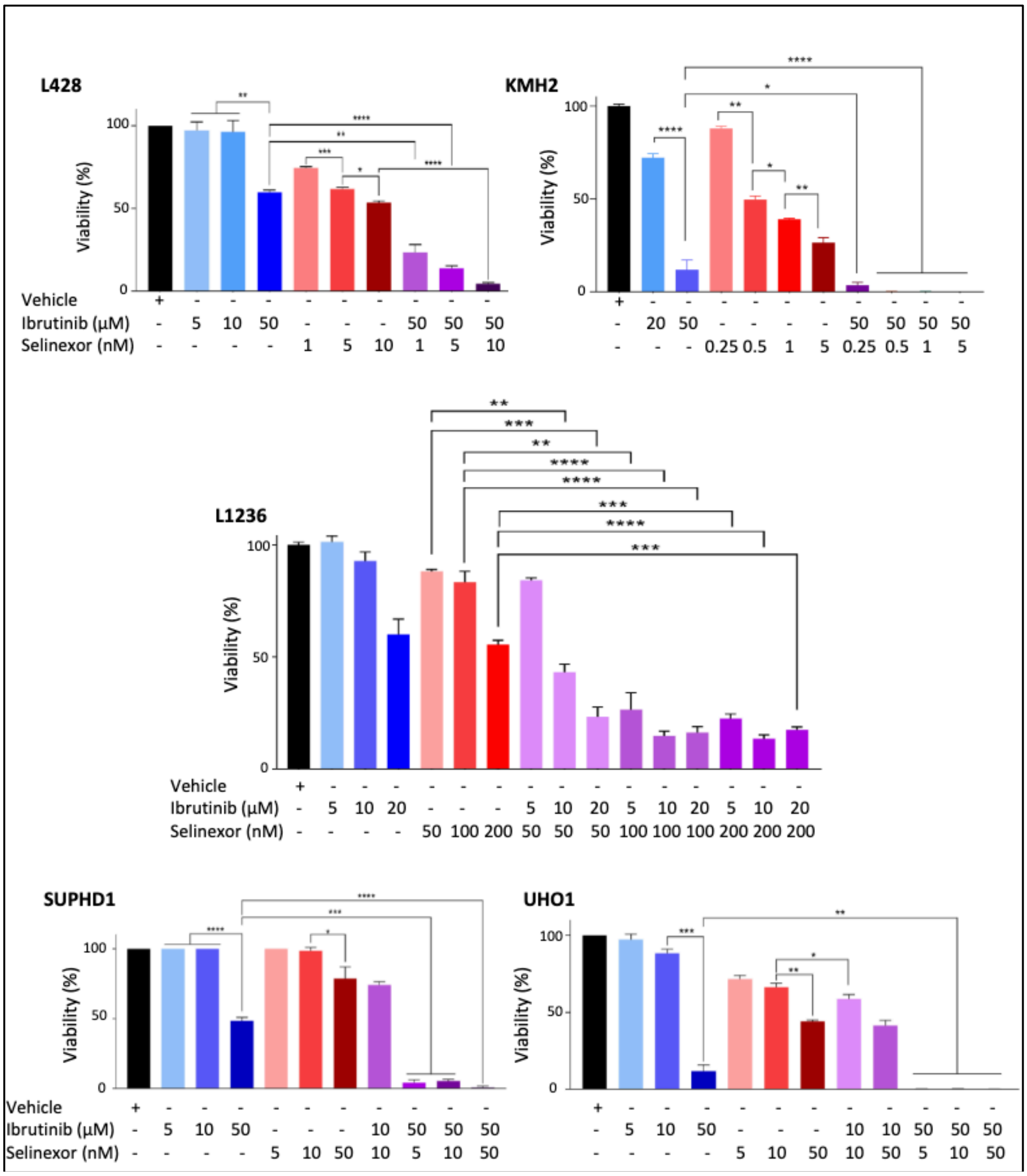

Fig. S9. The selinexor/ibrutinib combination is synergistic on cHL cells

cHL cells were seeded in 96-well plates at the density of 5 x 10^4 (KMH2, L428, L1236, and SUPHD1) or 7.5 x 10^4 (UHO1) cells per well then treated for 72 h with vehicle (0.01% DMSO), selinexor (Sel, 5 nM-10 µM, as indicated), ibrutinib (Ib, 5-50 µM, as indicated), or their combination. Cell viability was assessed by an MTS assay. On the graph are presented the means ± s.d. of one representative experiment performed with triplicate samples. The experiments was performed twice with similar results. The viability of cHL cell lines for each culture condition was calculated vs the control (referred to as 100%) by the Prism software (v8.0) and verified with the Compusyn software. * $p < 0.05$, ** $p < 0.01$, *** $p < 0.001$ and **** $p < 0.0001$ with the *t*-test.

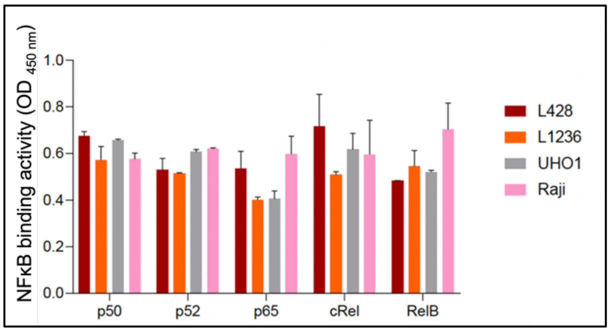

Fig. S10. DNA binding activity of NFκB proteins in cHL
The DNA binding activity of cHL nuclear protein extracts was quantified with an ELISA-based assay (TransAM NFκB Activation Assay Kit, Active Motif, Carlsbad, CA). Nuclear proteins were prepared from cultured cells using the Nuclear Extract Kit (Active Motif) then quantified. The assay was performed with 2 µg of nuclear proteins, each condition was set up in triplicate according to the manufacturer's protocol. The assay was run twice and analyzed using a multilabel plate reader (Victor X4, Perkin Elmer, Waltham, MA).The binding activity of each NFκB family member (p50, p52, p65, cREL, RELB) is reported on the histogram for L428, L1236 and UHO1 cHL cell lines as well as Raji cells, the internal positive control (means ± s.d.).

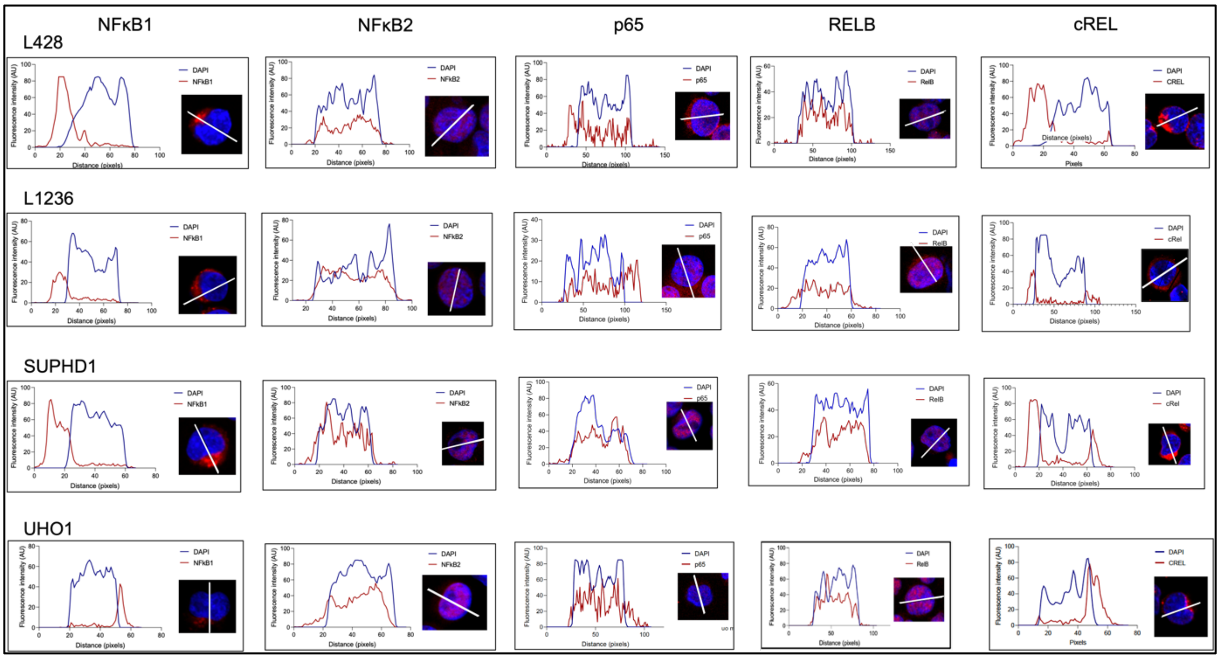

Fig. S11. Indirect immunofluorescence analysis of NFκB proteins in cHL cells
NFκB proteins expression was analyzed by IF in the four cHL cell lines. We used primary Abs (Table S1) and a goat Alexa Fluor 633-conjugated anti-mouse IgG or anti-rabbit IgG as secondary Abs. Slides were counterstained with DAPI and analyzed by confocal microscopy (x 180, magnification). Representative enlarged images (x 4) are shown. FIs of Alexa-633 fluorophore (in red) and DAPI (in blue) were estimated with the ImageJ software and data were exported to generate the curves of FI as a function of the distance along the white axis crossing the cell.

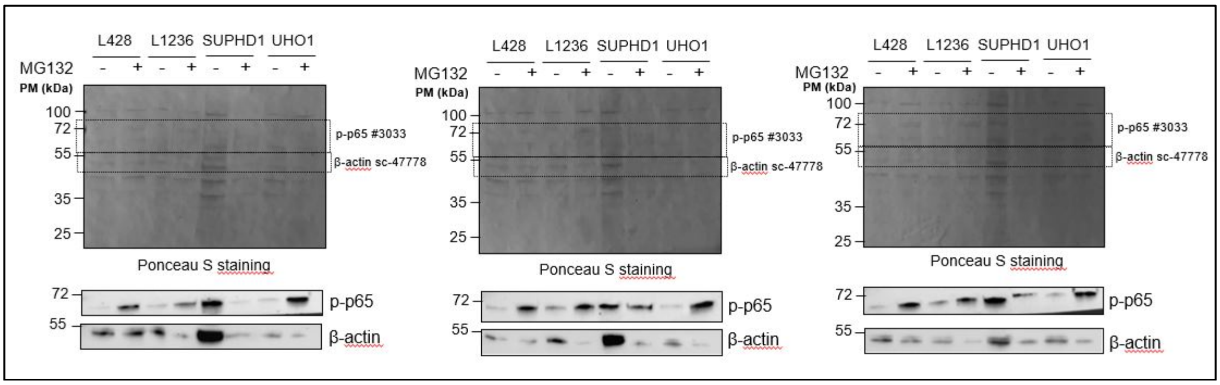

Fig. S12. Western blot analysis of p-p65 expression in cHL cell lines

Nuclear and cytoplasmic extracts were sequentially purified from cultured cHL cells treated with vehicle or MG132 (1 µM for 24 h). Cytosolic proteins (30 µg) were separated by SDS-PAGE, transferred onto nitrocellulose membranes. Membranes were cut and strips were then incubated with the indicated Abs (Table S2). Three independent blots were run and each blot was analyzed by densitometry for the quantification of p-p65 protein level relative to the β-actin level used as an internal control. The level of p-p65 in MG132-treated cells was then normalized to the one in vehicle-treated cells defined as 1. The values were used to draw the histogram presented in Fig. 6B and for the statistical analyses.

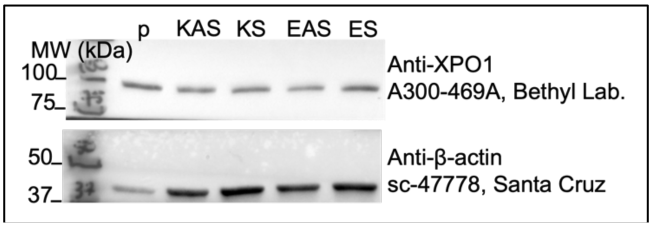

Fig. S13 - Original blots for Fig. 2C

Whole-cell proteins were purified, separated on SDS-PAGE, tranferred onto nitrocellulose membranes that were stained with Ponceau red. As presented membranes were cut into strips and incubated with the mentionned Abs (Table S1). Molecular weight markers (MW) were run in the same gels.
